# Effect of exogenous nitric oxide on sperm motility *in vitro*

**DOI:** 10.1186/0717-6287-47-44

**Published:** 2014-09-18

**Authors:** Jiangtao Wang, Qingliu He, Xingyu Yan, Youmei Cai, Junyi Chen

**Affiliations:** Departments of Urology, Affiliated Hospital, Shandong Medical college, Linyi, Shandong China; Departments of Urology, Second Affiliated Hospital, Fujian Medical University, Quanzhou, Fujian China; Departments of Ophthalmology, Second Affiliated Hospital, Fujian Medical University, Quanzhou, Fujian China

**Keywords:** Nitric oxide, Sperm capacitation, Sperm motility, Time, Human

## Abstract

**Background:**

Nitric oxide (NO) has been shown to be important in sperm function, and the concentration of NO appears to determine these effects. Studies have demonstrated both positive and negative effects of NO on sperm function, but have not been able to provide a clear link between NO concentration and the extent of exposure to NO. To study the relationship between nitric oxide and sperm capacitation *in vitro*, and to provide a theoretical basis for the use of NO-related preparations in improving sperm motility for *in vitro* fertilization, we investigated the effects of NO concentration and time duration at these concentrations on *in vitro* sperm capacitation in both normal and abnormal sperm groups. We manipulated NO concentrations and the time duration of these concentrations using sodium nitroprusside (an NO donor) and NG-monomethyl-L-argenine (an NO synthase inhibitor).

**Results:**

Compared to the normal sperm group, the abnormal sperm group had a longer basal time to reach the appropriate concentration of NO (*p* < 0.001), and the duration of time at this concentration was longer for the abnormal sperm group (*p* < 0.001). Both the basal time and the duration of time were significantly correlated with sperm viability and percentage of progressive sperm (*p* < 0.001). The experimental group had a significantly higher percentage of progressive sperm than the control group (*p* < 0.001).

**Conclusions:**

We hypothesize that there is a certain regularity to both NO concentration and its duration of time in regards to sperm capacitation, and that an adequate duration of time at the appropriate NO concentration is beneficial to sperm motility.

## Background

Infertility is a common clinical problem, affecting approximately 15% of couples, with a male factor influence in 30-60% of cases [1]. The effects of reactive oxygen species (ROS) and their importance in both physiological and pathophysiological events has been the subject of considerable study in recent years. Several ROS, including hydrogen peroxide (H_2_O_2_), superoxide anion, and nitric oxide (NO), have been shown to be involved in processes important to sperm physiology. Under normal, tightly regulated physiologic conditions, these ROS are essential to *in vitro* events necessary for the fertilizing ability of sperm [2]. Nitric oxide plays an important role in a variety of physiologic processes, including cellular information transmission [3], cellular defense [4], and as a regulator in both male and female reproductive functions [5]. The majority of evidence supports the view that at levels exceeding physiologic concentrations (generally considered to be less than one micromolar [6]), disruption of sperm function occurs, but that at low levels, NO is essential for sperm function. At physiologic levels, NO has been shown to be important in sperm capacitation [7–11] and acrosome reaction [8, 11, 12], in the maintenance of sperm motility [13], and may have an anti-apoptotic effect in sperm [11].

While some investigators have found no evidence supporting a detrimental effect of NO on sperm [14], the majority of evidence supports the notion that supraphysiologic concentrations of NO negatively affect sperm function. Salvolini et al. [15] provided evidence that increased nitric oxide synthase (NOS) activity and elevated tyrosine nitration may be contributory to the pathogenesis of idiopathic asthenozoospermia. A study by Weinberg et al. [16] showed that increased NO from addition of sodium nitroprusside (SNP, an NO donor), inhibited sperm motility and was correlated with NO-mediated inhibition of sperm cellular respiration. Other studies have provided evidence that elevated levels of NO decrease motility [17–23], usually in a concentration dependent [17, 18] and time dependent [18] manner, and are associated with increased sperm toxicity [17] and apoptosis [20].

However, although the physiologic effects on sperm modulated by NO appear to depend on both the concentration of NO and the duration of NO exposure [24], further clarification is needed regarding the relationship between the timing of NO exposure and the concentration of NO and sperm motility and capacitation. The present study collected both normal and abnormal semen samples for real-time monitoring of both NO concentration and time changes during sperm capacitation. Changes in these parameters were also monitored while artificially controlling the NO concentration with the addition of SNP and NG-monomethyl-L-arginine (L-NMMA, an NOS inhibitor), which allowed us to hypothesize on the relationship between NO concentration, time changes, sperm motility and sperm capacitation.

## Results

In the normal sperm group, the basal time (T1) needed to reach the appropriate NO concentration for capacitation was 32.09 ± 4.90 minutes. This concentration of nitric oxide (D) was 19912.33 ± 1359.95 nM, and the duration of time (T2) at this concentration of NO was 11.27 ± 2.42 minutes. In the abnormal sperm group, T1 was 79.46 ± 9.61 minutes, D was 19513.60 ± 1914.72 nM, and T2 was 31.89 ± 4.92 minutes.

**Figure 1:**
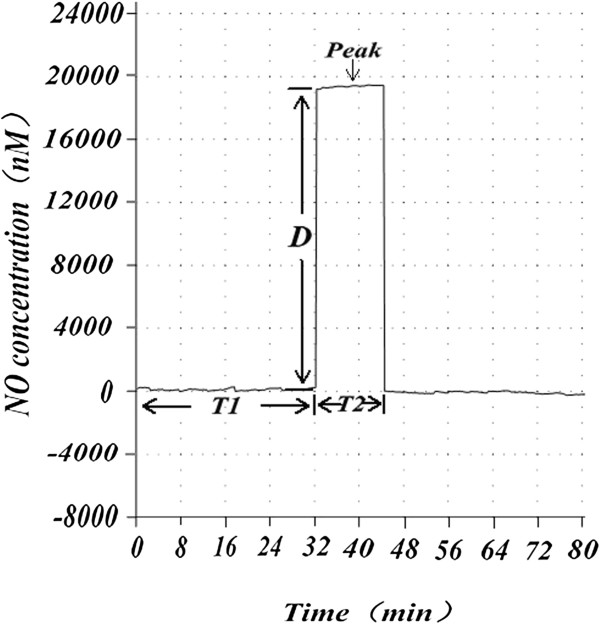
**Nitric oxide concentration and its time change in normal sperm capacitation** ***in vitro.*** The basal time T1 represents the length of time from the beginning of sperm capacitation to the NO concentration reaching an appropriate level. The duration of time T2 represents the duration of time at this NO concentration (D). Peak represents the appropriate NO concentration fluctuation. From Figure 1, we can see the relationship between changes in concentration of NO and time changes in the normal semen group.

**Figure 2:**
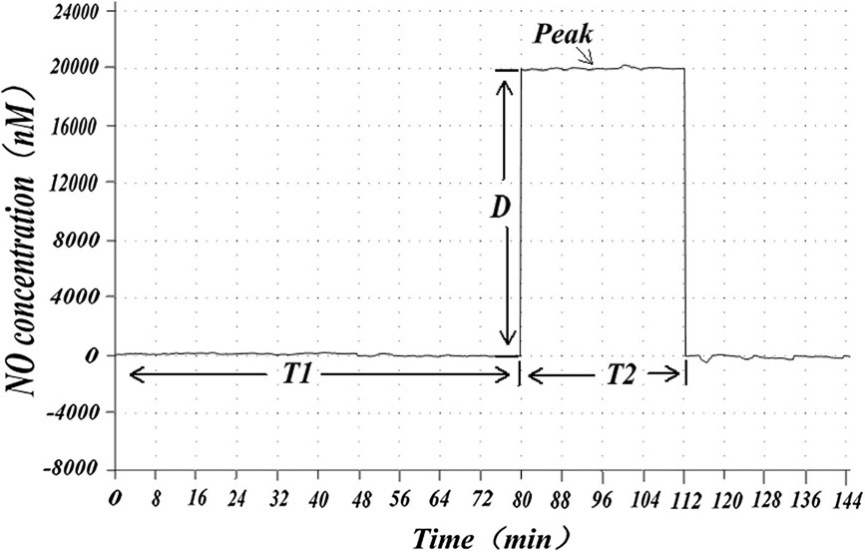
**Nitric oxide concentration and its time change in abnormal sperm capacitation** ***in vitro.*** T1, T2, and D as in Figure 1. In Figure 2, both T1 and T2 were longer in abnormal sperm than in normal sperm.

**Figure 3:**
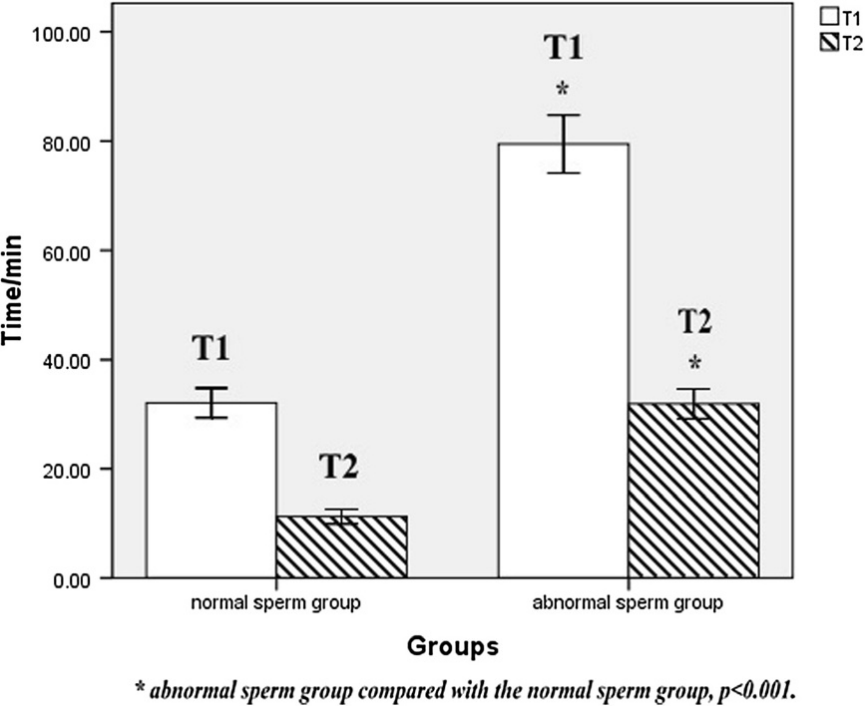
**The basal time** **(T1)** **and the duration of time** **(T2)** **in normal and abnormal sperm groups.** Results were expressed as mean ± standard deviation, n = 15. Both T1 and T2 of the abnormal sperm group were longer (*p* < 0.001) than that of the normal sperm group.

Based on the result of NO concentration over time during *in vitro* sperm capacitation in both the normal and abnormal semen groups, we reached the preliminary conclusion that, compared with the normal sperm group, the abnormal sperm group took longer to reach an appropriate NO concentration (t = −17.017, *p* < 0.001), and had a longer duration at these concentrations (t = −14.582, *p* < 0.001), but otherwise had no significant differences at these concentrations (t = 0.658, *p* = 0.516 > 0.05). These data are shown in Figures 1, 2 and 3.

**Figure 4:**
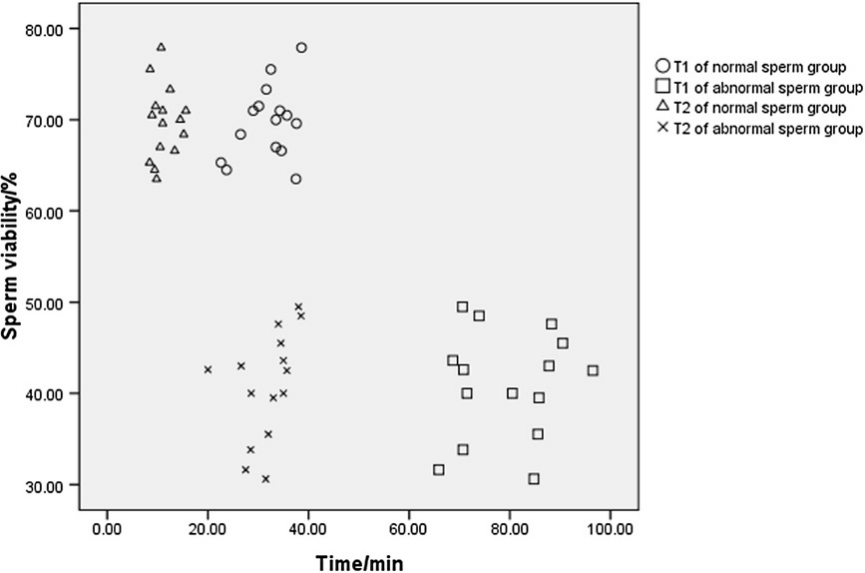
**The relationship between sperm viability and time in normal and abnormal sperm groups.** In this figure, the Y-axis represents the percentage of viable sperm in all samples and the X-axis represents the time (T1 or T2) in the normal and abnormal sperm groups. The correlation between sperm viability and T1 (r = −0.888, *p* < 0.001, n = 15) and between sperm viability and T2 (r = −0.853, *p* < 0.001, n = 15) was established through Pearson correlation analysis. Using the independent sample t-test, sperm viability in the abnormal sperm group was obviously lower than the normal sperm group (t = 15.598, *p* < 0.001, n = 15).

**Figure 5:**
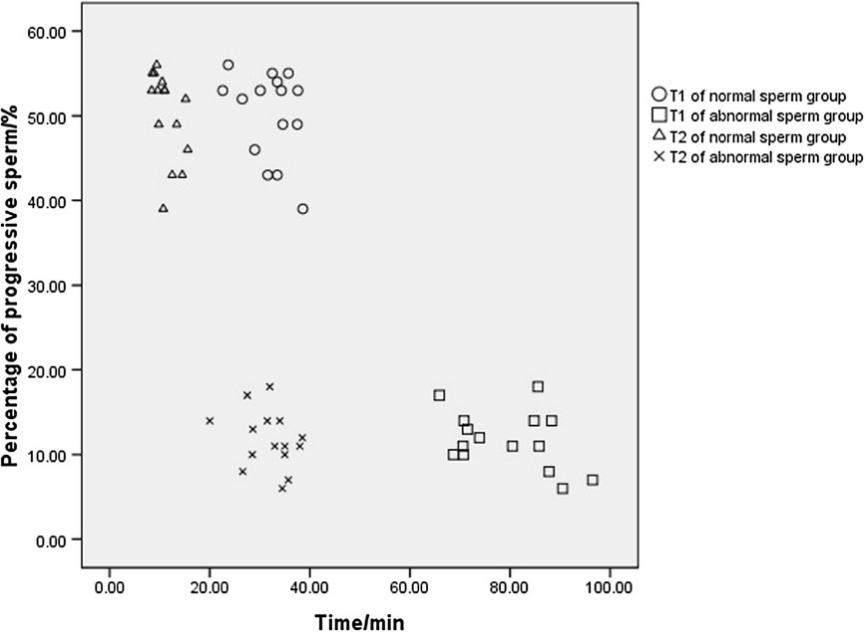
**The relationship between percentage of progressive sperm and time in normal and abnormal sperm groups.** In this figure, the Y-axis represents the percentage of progressive sperm in all samples and the X-axis represents the time (T1 or T2) in the normal and abnormal sperm groups. The correlation between the percentage of progressive sperm and T1 (r = −0.952, *p* < 0.001, n = 15) and between the percentage of progressive sperm and T2 (r = −0.942, *p* < 0.001, n = 15) was established through Pearson’s correlation analysis. Using the independent sample t-test, the percentage of progressive sperm in the abnormal sperm group was obviously lower than the normal sperm group (t = 24.003, *p* < 0.001, n = 15).

The T1s of the normal and abnormal sperm groups were significantly correlated with sperm viability (r = −0.888, *p* < 0.001) and the percentage of progressive sperm (r = −0.952, *p* < 0.001). The T2s of the normal and abnormal sperm groups were significantly correlated with sperm viability (r = −0.853, *p* < 0.001) and the percentage of progressive sperm (r = −0.942, *p* < 0.001). The NO concentration (D) of both groups had no obvious correlation with sperm viability (r = 0.113, *p* = 0.554 > 0.05) and percentage of progressive sperm (r = 0.120, *p* = 0.528 > 0.05). Compared with the normal sperm group, the abnormal sperm group had lower sperm viability (t = 15.598, *p* < 0.001) and a lower percentage of progressive sperm (t = 24.003, *p* < 0.001). These data are shown in Figures 4 and 5.

**Figure 6:**
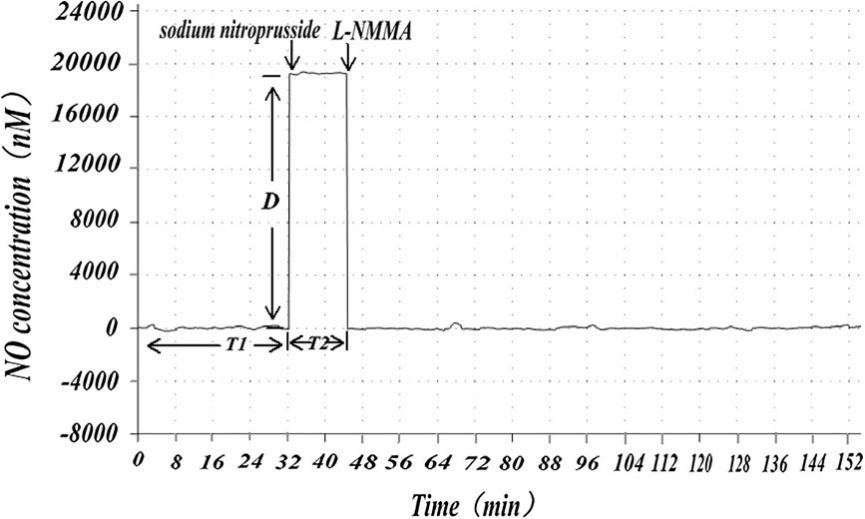
**The experimental group**’**s NO concentration and time change curve detected after adding SNP and L**-**NMMA.** T1, T2, and D as in Figure 1. SNP and L-NMMA were used to manipulate NO concentration in the experimental group, and, as seen in Figure 6, the experimental group’s NO concentration and time change curve is very similar to that of the normal semen group.

For the next part of the experiment, T1s of the control group (C1) and experimental group (C2) were 78.69 ± 8.92 minutes and 79.21 ± 9.12 minutes, respectively. Compared with C1, C2 showed no significant difference in T1. In the experimental group C2, SNP (100 nmol/L) was continuously added, beginning at thirty minutes after starting measurement of NO (the average time of normal sperm capacitation *in vitro*). There was an immediate increase in NO concentration, equivalent to the T1 peak in advance seen in Figure 6. This NO concentration was maintained by regulating the addition of SNP. When L-NMMA (10 mmol/L) was continuously added to C2, starting at forty-one minutes after starting measurement of NO (the point of average duration of normal sperm capacitation *in vitro*), the NO concentration rapidly returned to baseline levels, equivalent to T2 levels shorten. This NO concentration and time change curve is similar to the one seen in the normal semen group.

**Figure 7:**
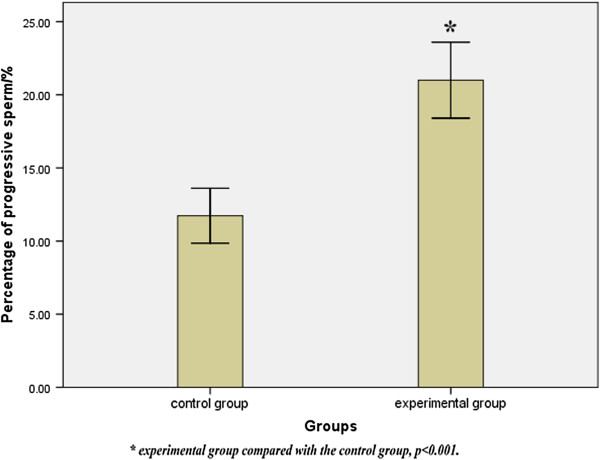
**Percentage of progressive sperm in the control and experimental groups.** Results were expressed as mean ± SD, n = 15. There was a statistically significant difference between the control and experimental groups in the percentage of progressive sperm (t = −6.201, *p* < 0.001).

By analyzing sperm quality parameters of the control and experimental groups, we found that the sperm viability in C1 was 40.92 ± 1.53%, the percentage of progressive sperm in C1 was 11.73 ± 0.88%, the sperm viability in C2 was 39.35 ± 1.43%, the percentage of progressive sperm in C2 was 21.00 ± 1.21%. The experimental group had a significant increase in percentage of progressive sperm (t = −6.201, *p* < 0.001), while both groups had no obvious difference in sperm viability (t = 0.746, *p* = 0.426 > 0.05). These data are shown in Figure 7.

## Discussion

The current evidence in the medical literature shows that ROS, including NO, are important in sperm function [5, 25, 26], and that, in the case of NO, the concentration appears to determine the effects on sperm motility [5]. As discussed earlier, studies have demonstrated both positive and negative effects of NO on sperm function, but have not been able to provide a clear link between NO concentration and the extent of exposure to NO.

Our present study sought to address this and was based on a continuous measurement of direct induction and manipulation of NO and observation of the time duration of NO concentration. Through real-time monitoring of the NO concentration change during sperm capacitation *in vitro*, our current experiment provides empirical evidence furthering the understanding of the relationship between sperm capacitation *in vitro*, sperm mobility, NO concentration and time changes. Compared to the normal semen group, the abnormal semen group had a longer basal time before reaching an appropriate NO concentration and remained at this concentration for a longer time. These time parameters were significantly correlated with both sperm viability and the percentage of progressive sperm. Elevated concentrations of NO-related enzymes (protein kinase A) can be detected 30 minutes after the initiation of sperm capacitation *in vitro*, suggesting that the change of NO concentration is most active at this point [27]. This most active time is approximately the same as the T1 of our normal semen group. An extended T1 in the abnormal semen group, seen as a time delay in the change of NO concentration reaching its most active point, could influence sperm viability and motility.

In addition, there were no obvious differences in the relative degree of NO concentration in both groups, and no significant correlation with sperm motility or other sperm quality parameters. Our findings support the assertion that sperm quality, particularly the percentage of progressive sperm as relates to capacitation [28], is related to the time needed to reach the concentration of NO appropriate for capacitation and the duration of time spent at this concentration of NO, rather than the concentration itself. These results are in accordance with the findings of du Plessis et al. [29], which also provided evidence that the degree of NO concentration has no significant correlation to semen quality parameters in sperm capacitation.

Several studies have examined the manipulation of NO concentration and its effect on sperm function. Nitric oxide donors have been shown to improve overall sperm quality [30]. Sodium nitroprusside has been shown to improve post-thaw sperm motility [31], improve motility and viability [32], inhibit sperm membrane oxidative damage [31, 32], and increase intracellular cGMP in both normal and asthenozoospermic semen samples [32]. Nitric oxide donors have also been shown to directly simulate the acrosome reaction in mouse sperm [33]. In other animal models, inhibition of NO synthesis decreases sperm motility and reduces hyperactivation in the late stage of capacitation [34], and inhibits the acrosome reaction [35].

Our present study provides further support for the effects of NO concentration of sperm function. Based on our results, we speculate that the sperm function of the abnormal semen group is affected by two factors: the delay in reaching an appropriate NO concentration in the early stage of sperm capacitation *in vitro*, and the extended duration of time spent at this NO concentration in the late stage of sperm capacitation *in vitro*. In addition, the persistence of NO appears to influence sperm viability, likely because the activation of NO-related enzymes in abnormal semen is slow than in normal semen, causing the sperm to be exposed to a certain NO concentration for a longer time, a condition that could have a detrimental effect on sperm progression and sperm capacitation *in vitro*. By manipulating NO concentration in the experimental abnormal sperm group (C2) using SNP and L-NMMA, we were able to maintain the NO concentration at a constant level, similar to that of the normal semen group. We were also able to reproduce a time change curve of NO concentration similar to that of the normal semen group, and in particular, were able to approximate the NO concentration of late stage sperm capacitation *in vitro*. Using this process, we were able to demonstrate a significant increase in the percentage of progressive sperm, and show that by controlling the NO concentration and time duration, we can improve both sperm motility and sperm capacitation *in vitro*. These findings suggest that clinical utilization of NO-related preparations may improve sperm motility and function, and thus conception rates, in infertile and subfertile men.

## Conclusions

By studying the mechanism of action of NO in sperm capacitation, we recognize there is certain regularity to both NO concentration and its time change in regards to sperm motility *in vitro*, and that these factors are conducive to capacitation. Based on our current results, further research into developing preparations of NO that can be used with *in vitro* fertilization to improve both sperm quality and fertilization rates may be considered. However, further basic work remains to be done to provide additional evidence to support the use of NO preparations in this capacity. Our experiment also has some limitations affecting the outcomes, such as the most effective timing and dosing of NO-related preparations, and the effects of the differences of the sperm capacitation process *in vitro* and *in vivo*. What we found from our present study are only preliminary results, and the safety of improving the sperm motility through regulation of the NO concentration should be further confirmed. While work in this area is quite complicated in both design and execution, we believe that a more detailed understanding of the action of NO will provide the basis for significant advances in the clinical treatment of male factor infertility.

## Methods

### Materials

Semen samples were obtained from the infertility clinic and outpatient urology clinic of the Second Affiliated Hospital in Quanzhou, Fujian, China. All participants were randomly selected and signed informed consent for the study. The normal semen samples (n = 15) were from men aged 23 to 31 years old (mean 25.5 years), and the abnormal semen samples (n = 15) were from men aged 24 to 30 years old (mean 24.9 years). Classification of the samples to the normal or abnormal groups conformed to the World Health Organization (WHO) semen testing standards [36]. Criteria for the normal group included semen liquefaction time < 30 minutes, sperm concentration ≥15 × 10^6^/ml, sperm viability ≥58%, and percentage of progressive sperm ≥32%. Criteria for the abnormal sperm group included semen liquefaction time > 30 minutes, sperm concentration <15 × 10^6^/ml, sperm viability <58%, and percentage of progressive sperm <32%.

### Semen analysis and sperm processing

Subjects were instructed to abstain from ejaculation for 3 to 5 days prior to sample collection. Semen samples were obtained via masturbation, collected in disposable sterile containers, and kept at room temperature until liquefaction occurred. Semen analysis was completed using a computer-assisted semen analyzer. Sperm were isolated from semen samples by using Percoll gradient centrifugation [37]. Sperm samples were incubated at 37°C for one hour in Earle’s balanced salt solution (SigmaAldrich, St. Louis, MO) [36, 38] before utilization.

### Nitric oxide detection

NO detection was carried out using the inNO-T nitric oxide measurement system consisting of an NO sensor and inNO System (Innovative Instruments, Inc., Tampa, FL), allowing the NO concentration to be measured by the NO sensor, with numeric values displayed simultaneously. The NO sensor was calibrated before each experiment. Our study group had successfully used this system previously to monitor the concentration of NO [39].

### Experimental procedures

We chose semen samples for the normal sperm group (group A, n = 15) and the abnormal sperm group (group B, n = 15) based on initial semen analysis. Sperm were obtained via Percoll gradient centrifugation and reached capacitation via sperm culture as described earlier. Changes in NO concentration during sperm capacitation were measured in real time for both groups A and B.

An additional group of abnormal semen samples (group C, n = 30) was randomly selected and divided into a control (C1) and an experimental (C2) group. Sperm processing methods were the same as with groups A and B, and NO concentration and time changes were measured *in vitro* while manipulating NO concentration. Thirty minutes after starting measurement of NO concentration (the average time of normal sperm capacitation *in vitro*), SNP (100 nmol/L) was continuously added to group C2, with a subsequent rise in and stabilization of NO concentration at around 19,000 nM-20,000 nM. At forty-one minutes (the average duration of time for normal sperm capacitation *in vitro*), L-NMMA (10 nmol/L) was added to group C2 until changes in the NO concentration returned to baseline. During the procedures, NO-related preparations were placed into containers connected with the infusion tube, and then the preparations were added at a constant rate, controlled by the regulating valve on the infusion tube. AmiNO-700 sensors were placed into the sperm samples, measuring the change of the NO concentration with an associated curve shown on the analyzer computer. The inNO-T nitric oxide measurement system was used for the in situ detection and real time measurement of NO. Sperm quality parameters were measured for both groups.

### Statistical analysis

We performed statistical analysis using SPSS 17.0 (IMB, Armonk, NY). Results are shown as mean ± standard deviation. Data correlation analysis was performed between groups, and in-group data were analyzed using the independent sample t-test and Pearson correlation analysis. We considered results to be significant at *p* < 0.05.

## Abbreviations

ROS: Reactive oxygen species
H_2_O_2_: Hydrogen peroxide
NOS: Nitric oxide synthase
NO: Nitric oxide
SNP: Sodium nitroprusside
L-NMMA: NG-Monomethyl-L-arginine
T1: The basal time (the time needed to reach the appropriate concentration of NO)
T2: The duration of time (at the appropriate NO concentration)
D: The concentration of NO
C1: The control group
C2: the experimental group.
